# A National Trauma-Informed Adverse Childhood Experience Screening and Intervention Evaluation Project

**DOI:** 10.3390/children12040453

**Published:** 2025-03-31

**Authors:** Karissa M. Luckett, Rachel Gilgoff, Molly Peterson, Aldina Hovde, Stephanie Pinney, Ruth S. Gubernick, Lisa M. Schafer, Monika Sanchez, Steven Kairys

**Affiliations:** 1 The Center for Youth Wellness, San Francisco, CA 94124, USA; rgilgoff@stanford.edu (R.G.);; 2 New Jersey American Academy of Pediatrics, East Windsor, NJ 08512, USA; 3 The Center for Community Health Evaluation, Seattle, WA 98101, USA; 4 Department of Pediatrics, Hackensack Meridian School of Medicine, Nutley, NJ 07110, USA; steven.kairys@hmhn.org

**Keywords:** trauma-informed care, toxic stress, resilience, adverse childhood experiences, screening, intervention, wellness, pediatric provider, practice, caregiver, adolescent

## Abstract

Background/Objectives: Adverse childhood experiences (ACEs) are traumatic childhood events that can disrupt neurologic, endocrine, and immune regulation and increase the risk for poor health outcomes. This Trauma-Informed ACE Screening and Intervention Evaluation (TASIE) Quality Improvement (QI) Project, ECHO, evaluated (1) pediatric provider knowledge of ACEs, toxic stress, and trauma-informed care principles, (2) implementation of ACE screening and clinical response in practice, and (3) patient and provider perspectives around benefits and challenges of ACE screening. Methods: From November 2021 to May 2024, three cohorts, totaling 46 pediatric practices across the U.S., participated in the TASIE Project, which included 2 h ACE training, eight monthly 75 min ECHO sessions, and monthly QI coaching. A mixed-methods approach was used to evaluate monthly data, while patient and provider surveys and provider focus groups were used to evaluate the program. Results: All 46 participating practices implemented ACE screening by the project’s conclusion. Of the patients eligible for ACE screening, over half were screened for ACEs during the program. Providers increased comfort with discussing ACEs and screening questions. During the first month, the practices were reported to have provided education to 56% of patients, and by the end of the project, this rate increased to 79% of patients. Overall, 97% of caregivers and 92% of adolescents screened agreed or somewhat agreed that it is important for providers to know about ACEs and toxic stress so they can offer better care. By the end of the project, for each cohort, providers reported that they were able to screen effectively and efficiently in routine practice and were more familiar with local resources.

## 1. Introduction

Adverse childhood experiences (ACEs) are difficult and stressful events in childhood, such as experiencing abuse, neglect, or household challenges including divorce or separation, intimate partner violence, or living with a caregiver with mental illness, substance misuse, or a history of incarceration. A higher number of ACEs can lead to the presence of toxic stress. Toxic stress may have implications on the body through the long-term effects of a chronically dysregulated stress response system [1]. The chronic dysregulation of the neuroendocrine immune system via the hypothalamic–pituitary axis (HPA) has been documented to negatively affect the immune, cardiovascular, reproductive, and endocrine systems [2,3]. In children and adolescents, ACEs have been associated with fair or poor general health [2,4], illness requiring a doctor [2], increased risk for infection [5,6,7], increased somatic complaints (e.g., headaches, nausea) [2], poor or disturbed sleep [8,9], fair or poor dental health [10], lifetime asthma [4,11], Attention Deficit Hyperactivity Disorder and autism [4], and overweight or obesity [4,12]. While the National Survey of Children’s Health has found that 34.8 million children across the United States are impacted by ACEs, many providers have not heard about ACEs and even fewer ask about ACEs in clinical practice [13,14]. A recent systematic review found that training medical providers about ACEs, ACE screening, and appropriate clinical response has been found to increase provider knowledge, confidence, screening uptake, motivational interviewing, and referrals. In addition, after provider training, patients reported greater satisfaction with their visit, a decrease in unmet needs, and were more comfortable discussing ACEs with their provider [15]. In 2021, the Center for Youth Wellness (CYW) partnered with the New Jersey Chapter, American Academy of Pediatrics (NJAAP), and the Center for Community Health Evaluation (CCHE) on a grant provided by the Health Resources and Services Administration (HRSA) of the U.S. Department of Health and Human Services (HHS) to launch the “Trauma-Informed ACE Screening and Intervention Evaluation (TASIE) Project ECHO^®^” Quality Improvement initiative. Using the Extension for Community Healthcare Outcomes (ECHO [9]) training model, three primary care practice cohorts (totaling 46 participating practices across the U.S.) were recruited, trained, and coached over a 9-month period to routinely screen children for ACEs, and provide interventions that build resilience, and appropriate referrals to community agencies. Cohorts were recruited after holding informational webinars about the project, which announced a Request for Proposal (RFP) process, which inquired as to practices’ knowledge, attitude, and behaviors related to ACE screening, as well as practice demographics, and other practice perceptions. The submitted RFP’s were then reviewed and scored by a team of staff from both CYW and NJAAP. The aims of this project were to (1) increase pediatric provider knowledge of ACEs, toxic stress, and trauma-informed care principles, (2) increase ACE screening and clinical response in practice, and (3) understand patient and provider perspectives on benefits and challenges of ACE screening.

## 2. Materials and Methods

From November 2021 through May 2024, a total of 46 primary care practices from 17 different states in the U.S. were divided into 3 cohorts (1 cohort per year) to participate in TASIE (Appendix A), a virtual model for teaching and supporting pediatric providers in screening for ACEs and providing relevant response and referral. Each of the selected 46 practices attended an orientation workshop, a 2 h ACEs training workshop, eight monthly 75 min ECHO sessions, and individual coaching sessions with one of the QI Coaches. Participating practices received a stipend between USD 10,000 and USD 15,000 to support their efforts. Practices were recruited via a publicly announced Request for Participation (RFP) and informational webinars, prior to each cohort. Between Cohort 1 and 2, the RFP was modified slightly to allow for a more thorough assessment of the requesting organization’s ability to make changes to their respective electronic health records (EHRs) as well as the organizational buy-in, and to have more than just one or two staff members participating in the program, as these were identified as barriers to successful participation in the initial cohort.

Screening tools: The Pediatric ACEs and Related Life-events Screener (PEARLS) tool was required for use to screen for ACEs, in its two available versions: (1) the de-identified or aggregate-level response format (responses recorded as a total count) or (2) the identified or item-level response format (responses were recorded as “yes” or “no” for each question). The tool is made up of two sections. Section one questions inquire about experiences of physical, sexual, and verbal abuse, emotional and physical neglect, and items of household instability such as mental illness, incarceration, intimate partner violence, substance abuse, and divorce. Section two of the tool focuses on community adversities and social determinants of health with questions about violence, discrimination, housing and food insecurity, parental/caregiver separation (through immigration issues or foster care), and parental/caregiver serious illness or death. The tool was also made available in over 15 languages, at the initial onset of the TASIE Project, and in the three reported versions: parent report for children, a parent report for teens, and teen self-report. The teen versions of the tool include a question in section two about romantic partner abuse as well. The total scores from section one and two are added together to provide the ACE score. This score, along with the presence or absence of physical symptoms of toxic stress, as assessed by a licensed provider, are used to determine the risk level for toxic stress in the individual’s life (Appendix B).

Clinical Response: Practices were provided training on the ACE screening clinical response based on level of risk for the presence of toxic stress and a set scoring algorithm (Appendix B). All patients and caregivers were to receive education on ACEs and toxic stress. In contrast, patients scoring in the intermediate- and high-risk categories would additionally receive anticipatory guidance, interventions, and referrals related to current Social Drivers of Health and one or more of the seven Domains of Wellness: Supportive Relationships, Quality Sleep, Balanced Nutrition, Physical Activity, Practicing Mindfulness, Experiencing Nature, Mental Health (also known as Stress Busters as part of the California ACEs Aware Initiative). The seven Domains of Wellness (DOWs) are a set of evidence-based strategies and interventions that reduce the effects of toxic stress and build resilience. DOWs are commonly addressed by pediatric providers in the context of the annual well-child visit as part of the provider’s anticipatory guidance for well-being and can be offered using a trauma-informed lens and motivational interviewing techniques [1,16,17].

Data Collection, Analysis, and Reporting Mechanisms: The evaluation used a mixed-methods approach to understand progress, facilitators, and barriers to implementing ACE screening and response in pediatric primary care. Data were collected monthly from practices using the Quality Improvement Data Aggregator (QIDA), an online project portal for data entry. Team leaders from each practice were provided with a secure user ID and password to access QIDA to enter and review their team’s data. The process implementation measures collected in QIDA included the percentage of eligible patients (1) screened, (2) scoring positive, (3) receiving referrals, and (4) declining screening. Collected patient demographic data allowed for stratification by different sociocultural groups, so that any significant differences among groups could be identified and evaluated. Data were collected from providers through SurveyMonkey^®^ pre/post participation and ECHO evaluation surveys. Data were collected from patients/caregivers through a REDCap 15.2.1 survey. In addition, iECHO, Project ECHO’s^®^ web-based partner relations management tool, was used to manage the Project ECHO^®^ QI program and track data on TeleECHO™ session participation for the ECHO Institute.

The evaluation was conducted by CCHE at the Kaiser Permanente Washington Health Research Institute (Seattle, WA, USA). A triad framework was used to evaluate the impact, with providers, patients/caregivers, and implementation process measures of the screening and intervention of ACEs. Within the triad areas, both qualitative and quantitative data were collected and analyzed.

## 3. Results

### 3.1. Types of Practices

During the three years of the TASIE Project, three cohorts of 46 diverse practices participated in the project: 17 practices in Cohort 1, 13 practices in Cohort 2, and 16 practices in Cohort 3. About half of the practices identified as an independent primary care practice (*n* = 25, 54%) and almost half served between 1000 and 4999 pediatric patients annually (*n* = 21, 46%). Six of these practices included medical residents as part of their practice team participants.

Of those practices that participated in the project, 71% of providers had been in practice for over 10 years and 51% for at least 20 years. Most of the participating providers indicated they were of non-Hispanic ethnicity (88%); 41% identified their race as White; 37% Asian; and 13% Black or African American. Eighty-two percent of the providers identified as female.

The practices served different types of communities: 47% were urban, 40% were suburban, and 13% were rural. Most practices identified themselves as independent (63%), although some practices identified as hospital-affiliated (16%), academic-affiliated (13%), or community-based (13%).

### 3.2. Patient Demographics

A total of 13,623 patients were eligible for ACE screening during the TASIE Project period. Each practice defined eligibility based on the patient’s age and visit type. Over half of the eligible patients (65%) were between 3 and 11 years of age. Eighteen percent identified as Black or African American, 36% identified as White, and about half (46%) identified their ethnicity as Not Hispanic/Latinx. It is also important to note that 28% of patients either declined to state their race or the practice did not have those data. There was very little difference in patients’ race and ethnicity across each of the cohorts.

Across the cohorts that participated in the TASIE Project, practices screened a variety of ages. Approximately half of participating practices screened children up to age 11, 17% screened ages 12 and older, and 15% screened children of all ages. All other practices screened select ages both under and over 12 (e.g., at well-child visits), but not all ages.

### 3.3. ACE Screening Implementation

**Figure 1:**
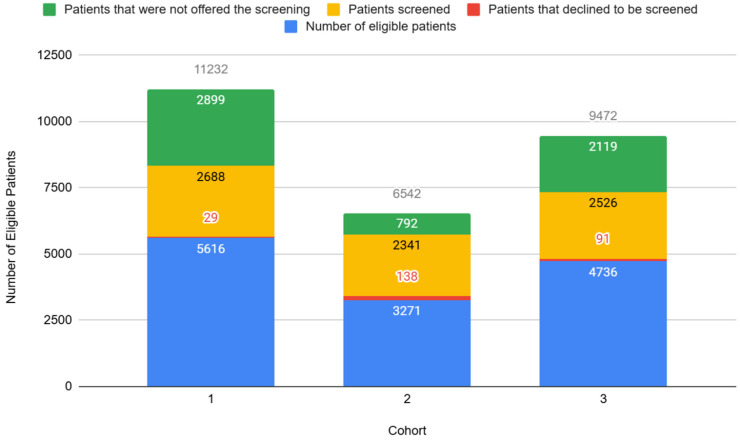
Eligible patients by screening status, *n* = 46 practices (source: QIDA).

**Figure 2:**
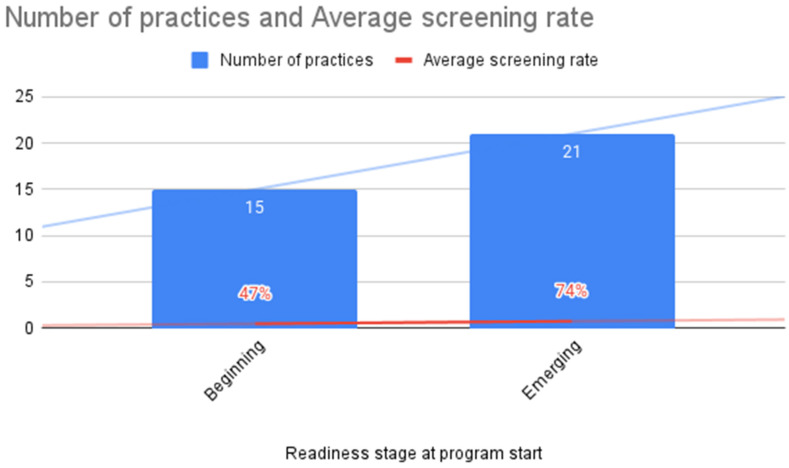
Average screening rate by practice starting readiness, *n* = 36 practices (Source: QIDA and provider survey). In total, 36 of the 46 practices had providers who responded to the pre- and post-survey with readiness ratings.

**Figure 3:**
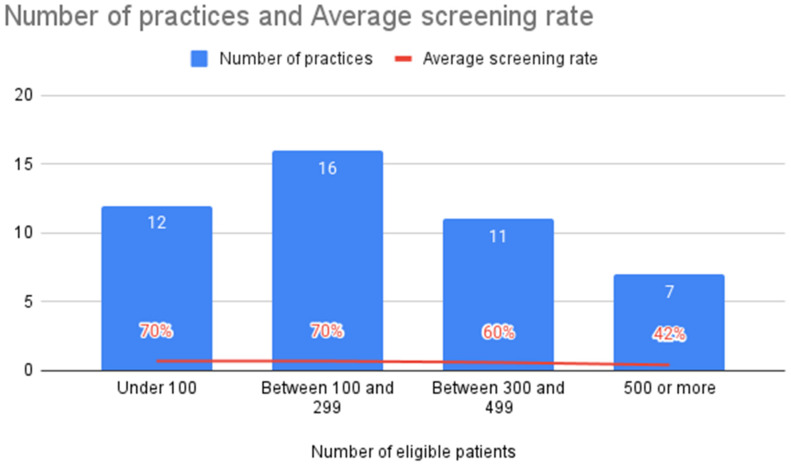
Average screening rate by eligible patient volume, *n* = 46 (source: QIDA).

Over half (*n* = 7555 patients, 55%) of the TASIE Project’s eligible patients were screened for ACEs during the program period (Figure 1). Practices who reported that they were in the lowest readiness stage at the beginning of the program were more likely to report lower average screening rates during the program (Figure 2). When practices reported that they started with a smaller sub-set of their target screening population—such as initially having all 3-year-old well-child visits on Mondays before expanding to all 3 year-old-patients with a well-child visit universally—the average screening rates appeared to be higher by the end of the program (Figure 3).

All participating practices in each cohort reported low rates of patients/caregivers who declined ACE screening; less than 2% for all eligible patients. In Cohort 2, one practice reported more than half of the declines. This practice attributed this high number of refusals to two things: (1) front desk staff not checking whether all the screenings were complete before the visit and (2) their electronic screening system. In Cohort 3, one practice reported their rate of declines were skewed upward due to miscoding, between declined cases and those not recorded for why they may not have been completed.

In all cohorts, approximately 75% of patients screened were low-risk, 13% intermediate-risk, and 9% high-risk for negative outcomes based on their PEARLS score. The distribution of risk status was consistent across cohorts.

The average number of providers per practice conducting screening in Cohorts 1 and 2 increased by about 1.4, from a little over 3 providers in the first data reporting cycle, to nearly 5 providers in the last data cycle. In Cohort 3, the average number of providers that reported participating in screening increased from almost 3 providers in the first cycle to nearly 9 in the final months.

### 3.4. Provider Knowledge

**Figure 4:**
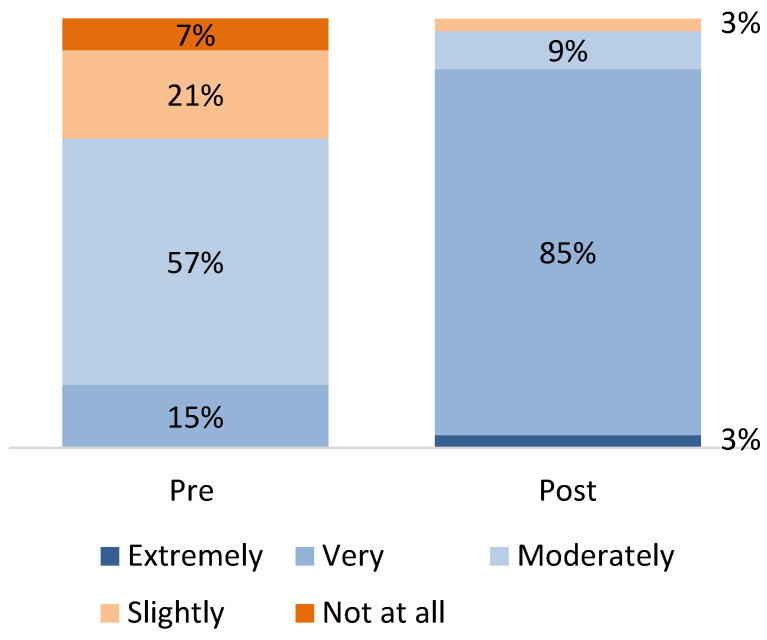
Knowledge of ACE screening and trauma-informed care, pre- and post-program (source: provider survey; *n* = 68 providers).

**Figure 5:**
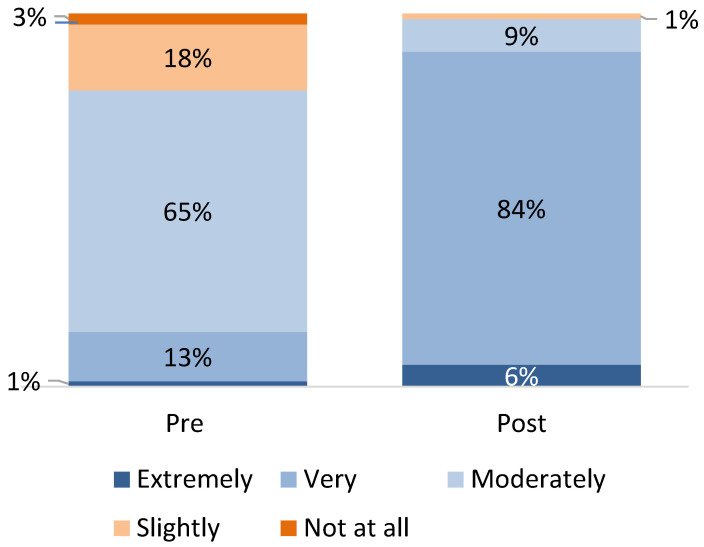
Knowledge of the science of ACEs and toxic stress and their impact on child health, development, and well-being, pre- and post-program (Source: provider survey; *n* = 68 providers).

Regarding knowledge of ACE screening and trauma-informed care, 85% of participating providers initially rated themselves as “Moderately” knowledgeable or less, shifting to 91% rating themselves as “Extremely” or “Very” knowledgeable at the conclusion of the program (Figure 4). Regarding knowledge of the science of ACEs and toxic stress and their impact on child health, providers went from only 13% feeling “Extremely” or “Very” knowledgeable, to 88% feeling this way by the end of the program (Figure 5). A few providers talked about this in the focus groups, noting that “There are 19 providers in our practice, and many of them had not heard of ACEs before. It was an eye opener for them to hear about and to learn about ACEs. I think that was really helpful”.

**Figure 6:**
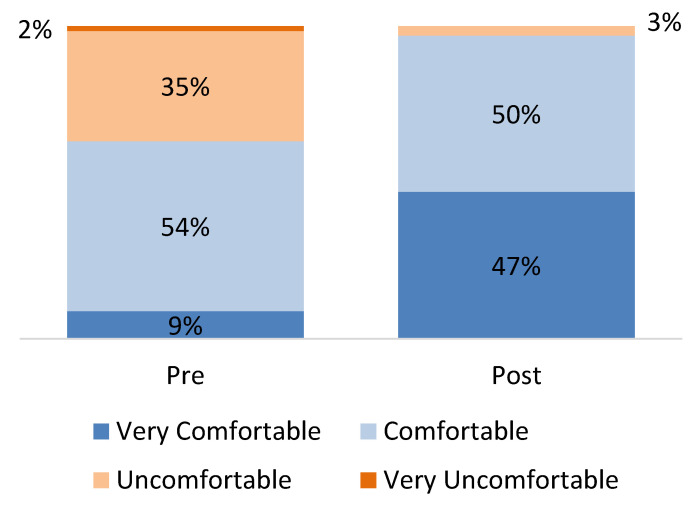
Comfort with discussion ACEs and ACE screening questions with patients, pre- and post-program (source: provider survey; *n* = 68 providers).

Providers were asked how comfortable they felt discussing ACEs and ACE screening questions with patients: 36% said they were “Uncomfortable” at the start of the program and by the end of the program, that reduced to just two providers (3%) (Figure 6).

In coaching conversations and focus groups, providers indicated that ACE screening helped them build empathy and better know their patients. “It’s a conversation starter. It reinforced that message: we care about your family, and this is just a continuation of all the things that we’re looking for in your child’s health—it’s not just the physical exam, there’s so many more aspects of it”. Another provider shared the following: “I think in the majority of situations the screening actually helps to build the therapeutic relationship with the patient and the family as a whole… It really opens up the discussion to recognize early a more vulnerable family and put a little extra effort on those topics as opposed to some of the other topics you have to cover in your child visits. I would say, don’t be afraid of the sensitive topics. Use it to build that therapeutic relationship early on with those families”.

### 3.5. Responding to ACEs

**Figure 7:**
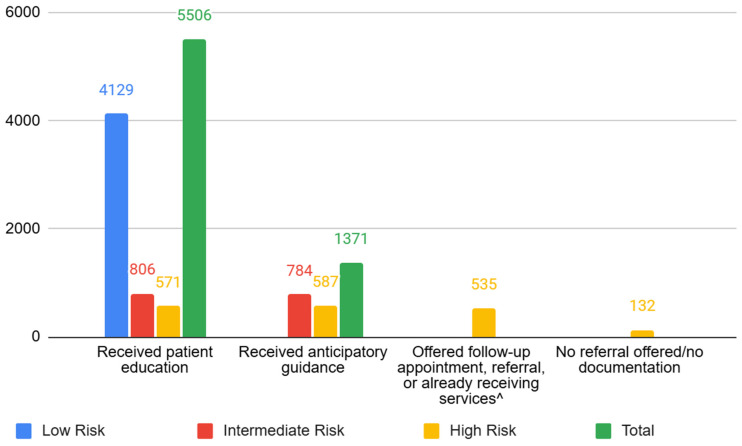
Number of patients with known risk status receiving/and or offered intervention by risk status, *n* = 7320 (Source: QIDA). Risk status was unknown for 196 patients; risk status was not reported/missing for 39 patients. Patients may receive more than one type of intervention.

A total of 7555 patients were screened as part of the TASIE Project. Of these patients screened, 7320 had a known risk status. Seventy-five percent of patients (*n* = 5506) across all known risk categories (low-, intermediate-, and high-risk) received patient education as recommended in the program algorithm (Figure 7). Providers in all participating practices across all cohorts increased their provision of patient education over time from about 56% of patients (at the beginning of the program), on average, receiving educational materials to 79% of patients receiving the same materials by the end of the project.

A total of 1663 patients scored intermediate- or high-risk; 82% of these patients received anticipatory guidance. A total of 667 patients scored high-risk; 80% of these patients reported speaking with their provider about receiving a referral or follow-up appointment. Some patients who scored high-risk were already connected to support (42%). During the monthly coaching calls with the practices’ designated coach, risk status and follow-up were discussed. Here, 18% of high-risk patients did not receive a referral, noted to be so due to a variety of factors such as follows: some practices not building in an option to document when patients were already in services; not having a specialist available in their area for some services (neuro-developmental pediatrician); and not documenting when a family refused a referral as being different from no referral given.

Typical referrals were for mental health (e.g., psychology/psychiatry, counseling, therapy), followed by other medical care specialties (e.g., neurology, ophthalmology), and other non-medical services (e.g., play therapy, housing, food pantry, community resources, social work). A few practices also reported external referrals to speech/occupational therapy and school-based services (e.g., Individualized Education Program).

In focus groups, providers perceived that the main barriers to referring patients to additional resources were the lack of availability (e.g., insufficient community resources, long wait lists, not accepting new patients), especially for mental health services, and insurance requirements. Some providers noted the difficulty of navigating complex referral systems, especially for families with limited English language proficiency.

To overcome these challenges, practices employed several strategies, including the following: (a) having a centralized list of resources; (b) leveraging local school district support for counseling; (c) building personal connections to services (e.g., taking a local therapist out to coffee); and (d) maintaining communication with patients while they were waiting to be seen by the other service providers.

In focus groups, many providers shared that using the Domains of Wellness (DOWs) with patients empowered them to know how to talk about ACEs with families. Providers reflected that the DOWs provided a framework to help patients and families identify free or easy-to-access supports (e.g., apps, parks, trails). In the absence of concrete referrals to offer, the DOWs gave something to work on or towards. “I was really hesitant when I saw the ACEs questionnaire. But the saving grace was being able to give those seven Domains of Wellness and feel like I had something to offer them after finding a positive score”. Another participant shared how “What was more helpful for the families was the framework [DOW] and to know they can do things on their own, because we also saw that for families, referrals could be a little overwhelming when we give a lot of them”.

**Figure 8:**
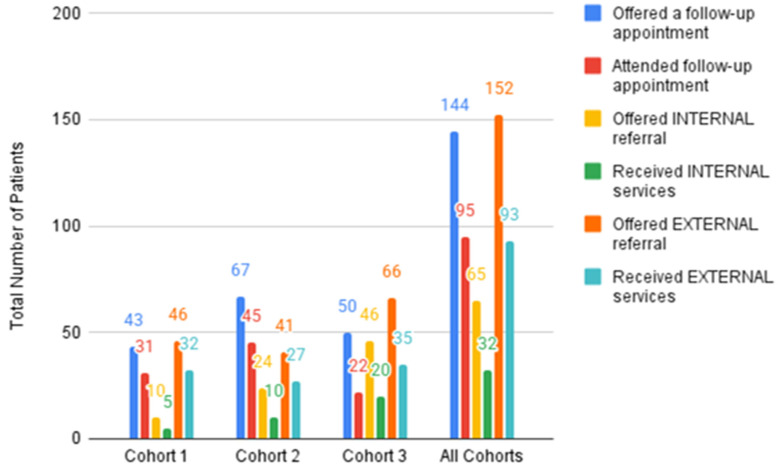
Number of known high-risk patients receiving/attending follow up or referral (source: QIDA). Note: Patients may receive more than one type of intervention. TASIE used Cohort 1 to pilot referral tracking and only practices with established referral systems tracked referrals and follow-ups. It is likely for this reason that the data show Cohort 1 with greater success in offering follow-up and patients accessing services. The evaluation defined an INTERNAL referral as an in-practice support like internal behavioral health, case managers, or care coordinator; EXTERNAL referral was defined as a support offered outside the practice, including community-based behavioral health providers, support for social needs (e.g., housing, food, or legal aid), or another organization/resource, including virtual resources (e.g., mindfulness apps).

Measures collected in QIDA accounted for referral outcomes, which were tracked for high-risk patients and allowed for an 8-week follow-up appointment window to transpire. Practices found that, on average, 66% of patients who received a follow-up appointment attended the appointment, 61% of patients received services from external referrals, and about half received services from internal referral sources (Figure 8).

### 3.6. Patient/Caregiver Experiences

On the patient and caregiver survey, 77% respondents recalled receiving the screening and 74% recalled talking about toxic stress with their provider. Of those surveyed, 51% of caregivers and 49% of adolescents reported that they learned new things about ACEs and toxic stress. Another 18% of caregivers and 35% of adolescents reported learning a lot of new things. As described by one respondent, the screening process “opened my eyes to things that I didn’t know”.

A few respondents mentioned concerns involving the sensitive nature of the topics, confidentiality, whether they would be judged or penalized for their responses, or how ACEs would be addressed. One caregiver commented, “I was a bit nervous answering the questions and hesitated to admit that I have had mental health issues in the past because it made me worry they’d try to take my kid away or something”.

**Figure 9:**
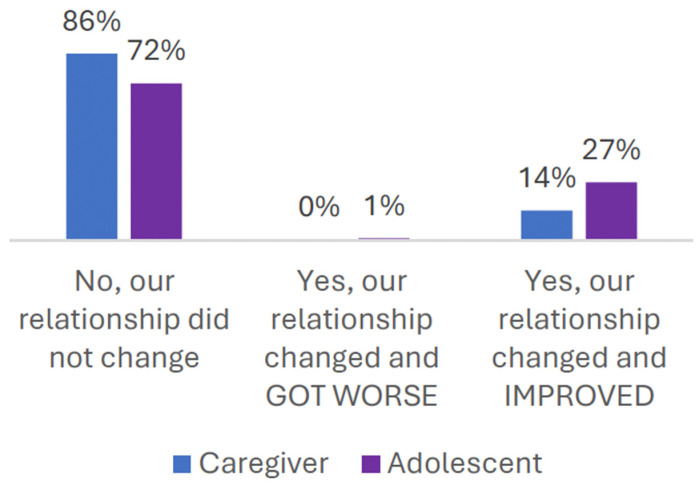
Relationship with provider after ACE screening (source: caregiver and adolescent survey, caregiver *n* = 210, adolescent *n* = 86).

Regarding the patient–provider relationship, 86% of caregivers and 72% of adolescents felt their relationship did not change after ACE screening, while 14% of caregivers and 27% of adolescents felt their relationship with their provider improved (Figure 9). Some respondents reflected feeling supported by the provider, noting that the conversation increased their comfort when talking about challenges and improved trust.

**Figure 10:**
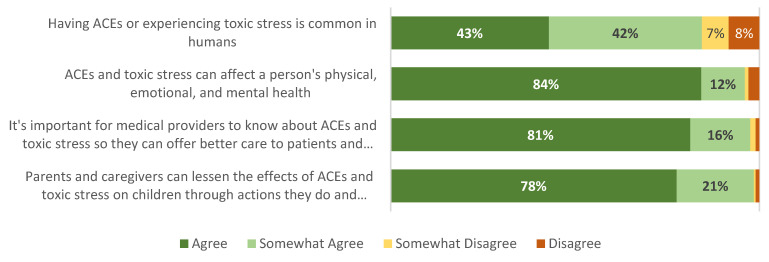
Caregiver knowledge and attitudes (source: caregiver survey, *n* = 210).

**Figure 11:**
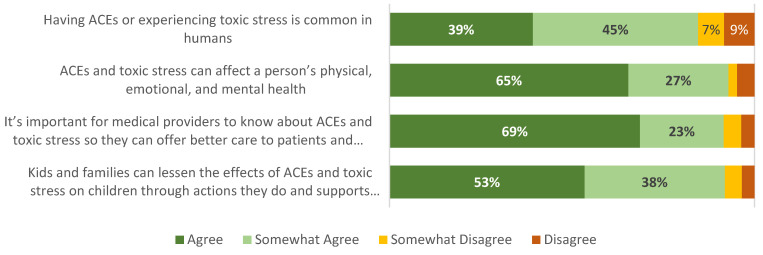
Adolescent knowledge and attitudes (source: adolescent survey, *n* = 86).

A majority of respondents agreed that (a) ACEs and toxic stress can affect a person’s physical, emotional, and mental health (84% of caregivers and 65% of adolescents); (b) it is important for medical providers to know about ACEs and toxic stress so they can offer better care to patients and families (81% of caregivers and 69% of adolescents); and (c) parents and caregivers can lessen the effects of ACEs and toxic stress on children through actions and support they have right now (78% of caregivers and 53% of adolescents). However, fewer than half of respondents agreed that ACEs or toxic stress is common (43% of caregivers and 39% of adolescents) (Figure 10 and Figure 11).

**Figure 12:**
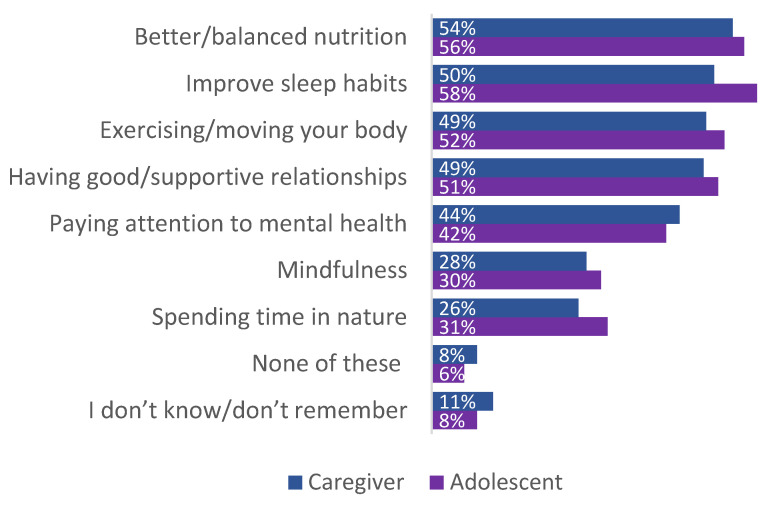
Information received about the various Domains Of Wellness (source: caregiver and adolescent survey; caregiver *n* = 210, adolescent *n* = 86).

Of caregivers and adolescents who responded to the patient survey, over 80% recalled receiving anticipatory guidance about one or more of the seven DOWs. Most often discussed were balanced nutrition, sleep, and exercising (Figure 12). A majority of caregivers (82%) and adolescents (74%) found the information on stress-mitigation strategies to be helpful or very helpful.

In Cohorts 2 and 3, the survey was modified to ask adolescents and caregivers to provide an example of a change they made related to the DOWs. Examples of changes reported by the forty-one caregivers who responded to the survey included earlier bedtimes and better sleep habits, going outside more regularly, altering the foods their family eats, and spending more quality time together as a family. A few mentioned increased efforts to listen to their children and improve communication. Ten adolescents responded to the survey and reported exercising more, implementing better sleep routines, eating better meals, and accepting themselves as they are.

Regarding referrals, among the nearly 300 caregivers and adolescents completing a post-visit survey, 70% of caregivers and 50% of adolescents reported not receiving a referral. This finding is consistent with the algorithm (Appendix B), which indicates offering additional services for those in the intermediate risk category and referring to trauma-informed therapeutic services and additional treatment as appropriate for those in the high-risk category.

Of those who reported receiving a referral (18% of caregivers and 28% of adolescents), 57% caregivers and 79% adolescents reported that they connected with the resource. Of those connecting with the resource (21 caregivers and 19 adolescents), 95% found it at least somewhat helpful.

## 4. Discussion

This evaluation reflects that the TASIE Project is a scalable model to help pediatric primary care providers effectively integrate ACE screening using a trauma-informed approach. The TASIE Project provided ACE screening tools, scripts, a clinical response algorithm, a toxic stress symptom checklist, patient education handouts, and strategies to mitigate the impact of toxic stress including the seven Domains of Wellness. This included clear guidance that ACE screening involved an ACE score alongside evaluation for current symptoms, ACE-associated health conditions, and protective factors. The TASIE Project was consistently and effectively implemented across three cohorts of different types of practices and patient populations across the country, leading to successful implementation of ACE screening in all participating practices.

This quality improvement (QI) project also demonstrated that training pediatric providers on ACE screening improved provider knowledge and practice and was well received by patients and families. By the end of each cohort, every practice was screening for ACEs, and providers were able to screen effectively and efficiently in routine practice and were more familiar with local resources. In addition, patients and caregivers overwhelmingly agreed that providers should know and talk about ACEs, and that it either did not change the patient–provider relationship or strengthened it.

Our evaluation found that two factors were associated with higher screening rates: organizational readiness reported before implementing ACE screening and the number of eligible patients to screen (a smaller number of eligible patients was associated with higher average screening rates). Geography, urban setting, presence of medical residents, size of annual pediatric population, age of eligible population, or choice of screener type (i.e., de-identified or identified PEARLS) did not appear to be associated with screening rates. These findings are similar to the existing literature describing how effective ACE screening is supported by foundational trauma-informed practices and capacities, including organizational leaders providing the resources for screening implementation (e.g., technology, staffing, training); providing education or training to all staff and providers on trauma and resilience and implications for care; and defining roles, responsibilities, and workflows for care team members related to screening processes [18].

Implementation facilitators, as reported by providers in focus groups and coaching sessions, included (1) providing resources and support as part of the TASIE Project, e.g., scripts, (2) building buy-in and screening implementation knowledge through staff and provider training, (3) having previous experience conducting pediatric screenings, e.g., developmental screenings, (4) prioritizing trauma-informed care, (5) identifying a champion for implementing screening and for advancing trauma-informed care approaches within the practice, (6) establishing internal and external resources and services (e.g., behavioral health supports), and (7) building trusting relationships with patients and families. While providers recognized the need to have resources and a referral network established prior to starting ACE screening, they also noted that they could not anticipate all of the resources and services needed. ACE screening helped expand their support services to include more stress-mitigation strategies, community connections, and behavioral support.

From our focus groups and interviews with providers, we found that ACE screening has led to (1) de-stigmatization and normalizing of trauma, (2) support for providers and patients to “open up” around physical and mental health issues, (3) the identification of patient needs that would otherwise have gone undetected, and (4) an opportunity for a strengths-based approach allowing for conversations with patients and families about their current positive activities, strengths, support, and resiliency. One provider noted that “Our office has been able to learn a lot more about our patients, even patients that have been coming here since birth. By using the screener, we uncover backstories from them and past traumas that would not have been found had we not had that screener, because there’s no fit for it unless the patient brings it up. So, we’ve been able to really help them through”. Providers found that having scripts and information on the Domains of Wellness were particularly helpful in improving comfort with discussing ACEs and ACE screening, and being able to provide patient education.

Our findings are similar to previous studies. Multiple studies have shown that ACE screening is feasible and useful in a variety of primary care settings, including prenatal care, pediatrics, family medicine, and adult primary care [19,20,21,22,23,24,25,26,27,28,29,30,31]. These studies suggest that ACE screening does not significantly increase visit times and may be associated with improved healthcare utilization. Educating patients about the link between adversity and health is often appreciated by patients and improves the patient–provider relationship. ACE screening in a trauma-informed medical practice has been appreciated by patients who can be offered supportive links to needed services [20,22,32]. Pediatric providers offer a unique opportunity for early detection and intervention to prevent the intergenerational transmission of adversity [33]. Pediatricians see children at regular intervals, are trained to provide anticipatory guidance to prevent and educate families about a wide variety of public health issues, and understand the important role of parents and communities in determining a child’s well-being [34,35]. Studies show that caregivers are open to ACE screening and are often unaware of the significant impact ACEs can have on the developing brain of a young child [16,26]. In addition, several states have now adopted ACE screening initiatives and provide payment for conducting ACE screenings to help support practices in responding to ACEs.

## 5. Limitations

The main limitation is the short period of time of the ECHO model, which provides 9 months of education and evaluation for each cohort. Thus, we could not capture long-term changes in practice transformation, provider or patient behavior, or patient health outcomes. While we found increases in provider knowledge and note that all clinics were successfully screening for ACEs by the end of the program, we were not able to capture long-term sustainability. Also, while our findings suggest that patients adopted behavior changes related to stress-mitigation strategies such as improved nutrition, sleep, and relational strategies, we could not assess long-term impacts to health.

To understand the relationship between ACE interventions provided at a child’s primary care visit and the impact on adult health outcomes, longitudinal studies outside the scope of this study would be necessary. Future research of child health outcomes could help answer questions that remain, such as the following: (1) did the intervention(s) provided reduce the effects of any ACEs the child currently has?; (2) did the intervention(s) prevent future ACEs from occurring; and (3) did the intervention(s) prevent or lessen any future negative health outcomes that would occur as a result of their ACEs?

## 6. Conclusions

Overall, we found that training providers on ACE screening can improve knowledge, confidence, and screening behavior; that ACE screening is feasible and acceptable to patients and caregivers; and that ACE screening can be performed in a supportive, strengths-based, trauma-informed manner, providing patients with stress-mitigation strategies and needed resources and referrals. We found the TASIE model to be a scalable educational tool to support pediatric primary care providers to effectively implement ACE screening and clinical response in a strengths-based and trauma-informed way. In the context of the existing literature, this work continues to reinforce that screening for ACEs is feasible and acceptable to both patients and clinicians in diverse geographic and clinic settings, offering a model for an ACE screening education and implementation program that could inform future clinical practice and research directions.

Early identification and intervention of toxic stress can shift the focus from just treating symptoms to understanding the source of health problems. Utilizing ACE screening and intervention helps providers have a more thorough history and can create a stronger patient–provider attachment that facilitates a true home medical experience, where individuals go to their outpatient provider for help when they are not feeling well, and when they want to partner on their health and well-being. A move to more of this proactive relationship between patient and provider could dramatically shift our health utilization, provide better well-informed care, and help to prevent long-term and chronic health condition development and exacerbation.

This project is supported by the Health Resources and Services Administration (HRSA) of the U.S. Department of Health and Human Services (HHS) as part of an award totaling USD 960,000 a year, for 3 years, with no percentage financed with non-governmental sources. The contents are those of the author(s) and do not necessarily represent the official views of, or an endorsement by, the HRSA, HHS, or the U.S. Government. For more information, please visit HRSA.gov.

## Figures and Tables

**Figure A1:**
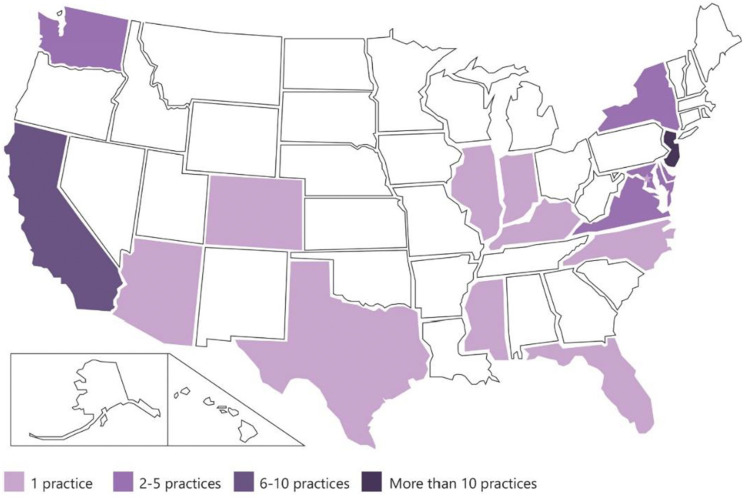
States with practices participating in the TASIE Project, Cohorts 1-3, *n* = 46 (source: enrollment data).

**Figure A2:**
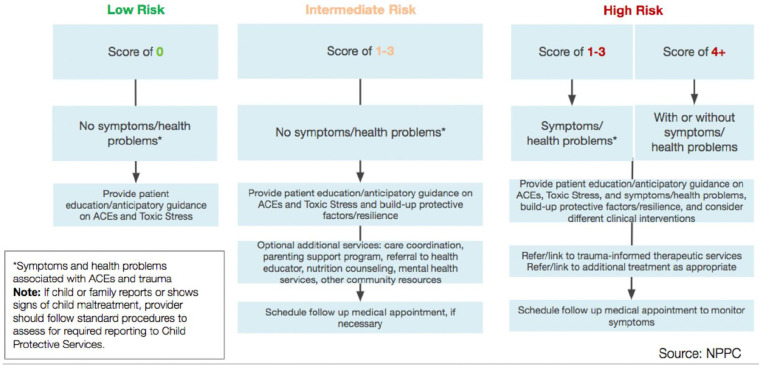
TASIE Project clinical response algorithm (Source: TASIE Implementation Guide).

## Data Availability

The original contributions presented in this study are included in the article. Further inquiries can be directed to the corresponding author.
